# Supplementary material for: Diverging co-translational protein complex assembly pathways are governed by interface energy distribution
Source: Nat Commun. 2024 Mar 25;15:2638. doi: 10.1038/s41467-024-46881-w (PMC10963368; doi:10.1038/s41467-024-46881-w)
Supplement: Supplementary file 1 — Supplementary Information [file 41467_2024_46881_MOESM1_ESM.pdf]

Supplementary Information

Diverging co-translational protein complex assembly pathways are governed by interface energy distribution

Johannes Venezian, Hagit Bar-Yosef, Hila Ben-Arie Zilberman, Noam Cohen, Oded Kleifeld, Juan Fernandez Recio, Fabian Glaser & Ayala Shiber

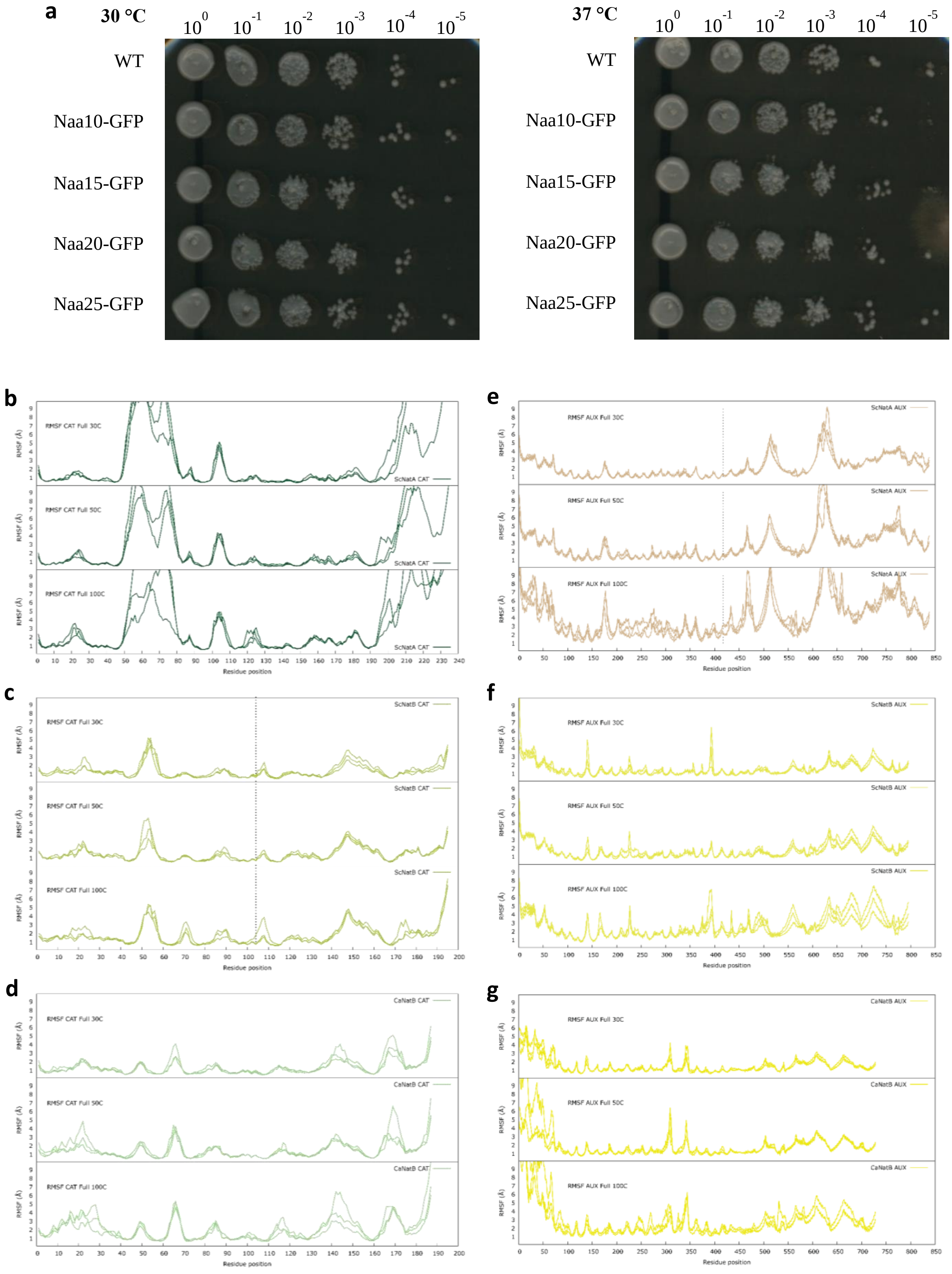
a
30 °C
10^{0} 10^{-1} 10^{-2} 10^{-3} 10^{-4} 10^{-5}
WT
Naa10-GFP
Naa15-GFP
Naa20-GFP
Naa25-GFP
37 °C
10^{0} 10^{-1} 10^{-2} 10^{-3} 10^{-4} 10^{-5}
WT
Naa10-GFP
Naa15-GFP
Naa20-GFP
Naa25-GFP
b
RMSF CAT Full 30C
RMSF CAT Full 50C
RMSF CAT Full 100C
ScNatA CAT
RMSF (Å)
Residue position
c
RMSF CAT Full 30C
RMSF CAT Full 50C
RMSF CAT Full 100C
ScNatB CAT
RMSF (Å)
Residue position
d
RMSF CAT Full 30C
RMSF CAT Full 50C
RMSF CAT Full 100C
CaNatB CAT
RMSF (Å)
Residue position
e
RMSF AUX Full 30C
RMSF AUX Full 50C
RMSF AUX Full 100C
ScNatA AUX
RMSF (Å)
Residue position
f
RMSF AUX Full 30C
RMSF AUX Full 50C
RMSF AUX Full 100C
ScNatB AUX
RMSF (Å)
Residue position
g
RMSF AUX Full 30C
RMSF AUX Full 50C
RMSF AUX Full 100C
CaNatB AUX
RMSF (Å)
Residue position

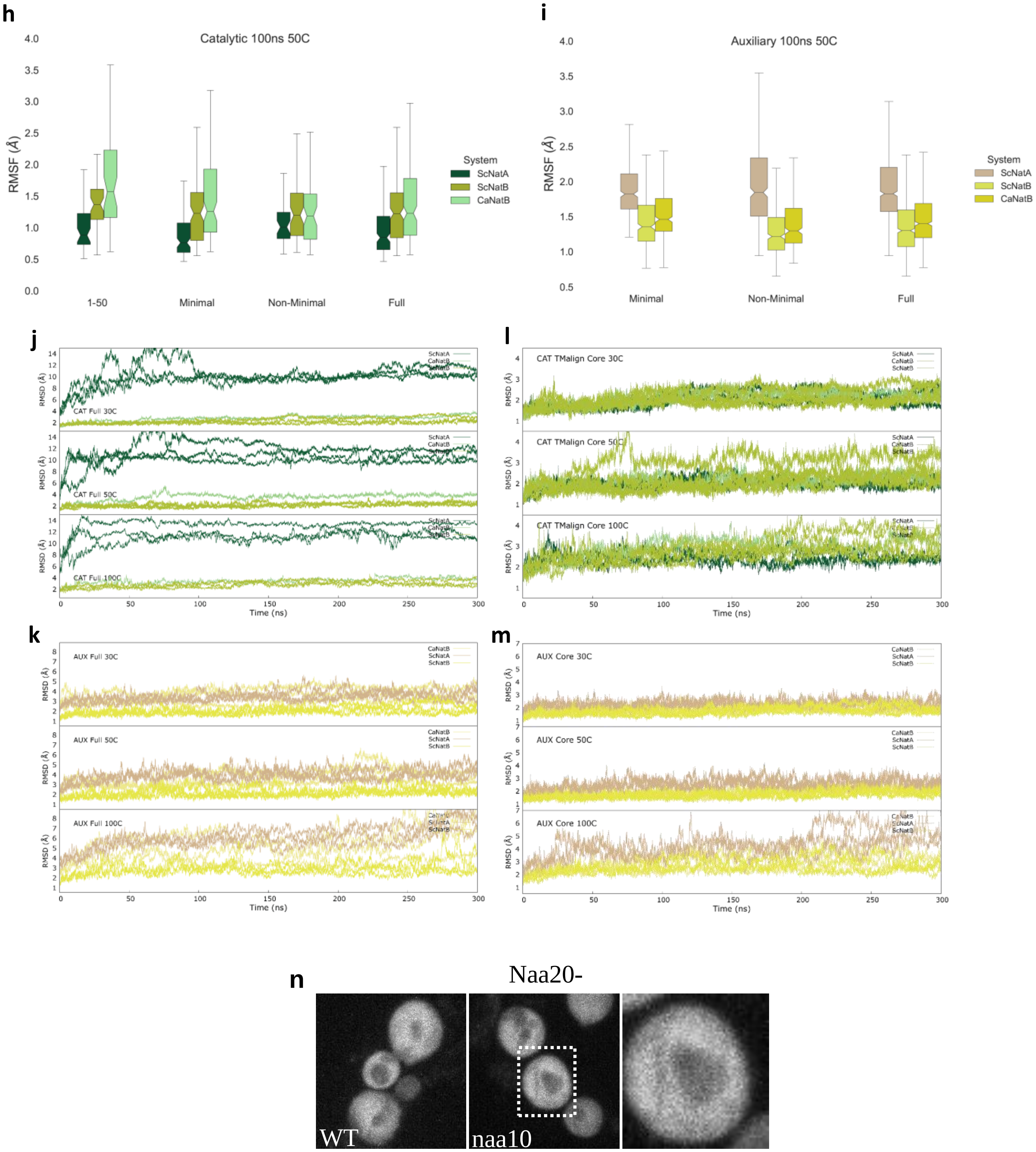
h
Catalytic 100ns 50C
RMSF (Å)
1-50
Minimal
Non-Minimal
Full
System
ScNatA
ScNatB
CaNatB
i
Auxiliary 100ns 50C
RMSF (Å)
Minimal
Non-Minimal
Full
System
ScNatA
ScNatB
CaNatB
j
CAT Full 30C
CAT Full 50C
CAT Full 100C
RMSD (Å)
Time (ns)
l
CAT TMalign Core 30C
CAT TMalign Core 50C
CAT TMalign Core 100C
RMSD (Å)
Time (ns)
k
AUX Full 30C
AUX Full 50C
AUX Full 100C
RMSD (Å)
Time (ns)
m
AUX Core 30C
AUX Core 50C
AUX Core 100C
RMSD (Å)
Time (ns)
n
Naa20-
WT
naa10

Supplementary Figure 1: NatA and NatB subunits` functionality and structural analysis

(a) GFP tagging does not affect the growth of the NatA and NatB subunits in *Saccharomyces cerevisiae*. Growth assay on YPD at 30 °C and at 37 °C of the indicated strains vs. wildtype (WT). All the tagged strains show no impact on growth. n = 3, a representative image is shown.

(b-d) RMSF of the free catalytic subunits of *Saccharomyces cerevisiae* NatA and NatB and *Candida albicans* NatB. All subunits were simulated at 30 °C, 50 °C, and 100 °C for 300 ns (x3 repeats). At all temperatures, the higher mobility of co-translationally dependent subunits (Naa20) remained, excluding Naa10's long, polar loops.

(b) RMSF of *Saccharomyces cerevisiae* NatA catalytic subunit at 30 °C, 50 °C, and 100 °C.

(c) RMSF of *Saccharomyces cerevisiae* NatB catalytic subunit at 30 °C, 50 °C, and 100 °C.

(d) RMSF of *Candida albicans* NatB catalytic subunit at 30 °C, 50 °C, and 100 °C.

(e-g) RMSF of the free auxiliary subunits of *Saccharomyces cerevisiae* NatA and NatB and *Candida albicans* NatB. All subunits were simulated at 30 °C, 50 °C, and 100 °C for 300 ns (x3 repeats). At all temperatures, the higher mobility of co-translationally dependent subunits (Naa15) remained.

(e) RMSF of *Saccharomyces cerevisiae* NatA auxiliary subunit at 30 °C, 50 °C, and 100 °C.

(f) RMSF of *Saccharomyces cerevisiae* NatB auxiliary subunit at 30 °C, 50 °C, and 100 °C.

(g) RMSF of *Candida albicans* NatB auxiliary subunit at 30 °C, 50 °C, and 100 °C.

(h) RMSF boxplots of the different regions of the catalytic and auxiliary subunits at 50 °C for 300 ns (x3 repeats). The regions in the catalytic subunits boxplot **(h)** include amino acids 1-50, the minimal region (before the onset of co-translational interactions), the post-minimal region, and the complete protein. For the auxiliary subunits boxplot (**i**), the included regions are the minimal region (before the onset of co-translational interactions), the post-minimal region, and the complete protein. Minimal regions are determined by co-translational interaction onset in *S. cerevisiae* and its equivalent in *C. albicans* as calculated by TM-Align.

(j-m) RMSD of the full-length subunits of the catalytic subunits (**i**) and auxiliary subunits (**j**) at 30 °C, 50 °C, and 100 °C for 300 ns (x3 repeats), as well as RMSD of the TM-Aligned cores of the catalytic (**k**) and auxiliary subunits (**l**) at 30 °C, 50 °C, and 100 °C for 300 ns (x3 repeats).

(n) NatA and NatB subunits solubility analysis, determined by localization patterns changes. Endogenously tagged Naa20-GFP *Saccharomyces cerevisiae* cells were grown to Log-phase (30 °C). Cells were then fixed and subjected to confocal microscopy. Representative images are shown. Scale bar, 4 µm. No significant aggregation of GFP tagged Naa20 (Catalytic subunit, NatB) was observed in either wildtype or *naa10*Δ (Catalytic subunit NatA complex deletion) strain. n > 150 cells.

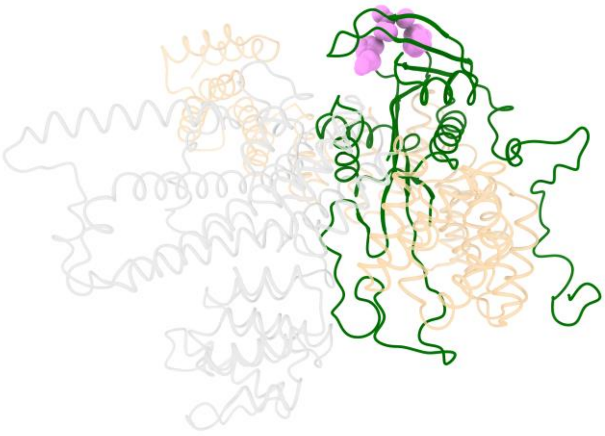

Supplementary Figure 2: TRiC/CCT chaperone binding sites.
As pink spheres, TRiC/CCT chaperone binding sites of *Saccharomyces cerevisiae* Naa10 (as green ribbon), as reported in Stein et al. (2019)[1]. Naa15 is represented as tan ribbon (minimal region) and grey ribbon (post-minimal region).

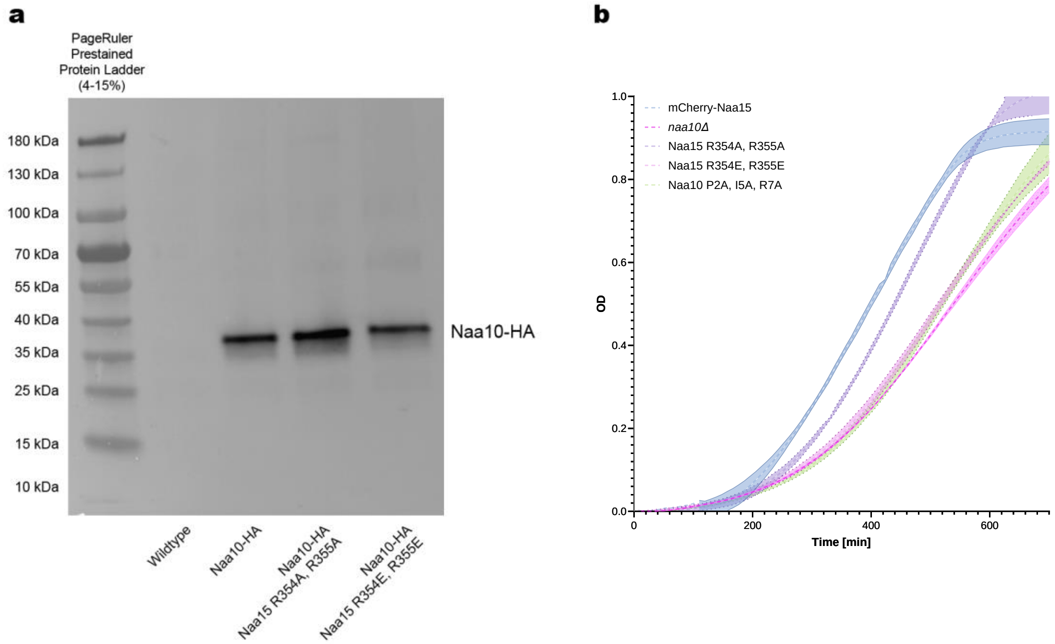

Supplementary Figure 3:
(a) Western blot analysis of immunoprecipitated HA-tagged Naa10**.** Western blot against HA (rabbit) after IP against HA (mouse, IgG2a). 10% of the IP product was used for Western blot while the rest was used to continue the RIP-qPCR. The ladder used is the Thermo Scientific PageRuler™ Prestained Protein Ladder.
(b) Growth curves analysis of the indicated mutated strains. Averaged OD_{595} of three biological replicates, each representing a growth curve of one strain. In dashed line is the mean and the degree of experimental variation (standard deviation) is shaded in the corresponding color.

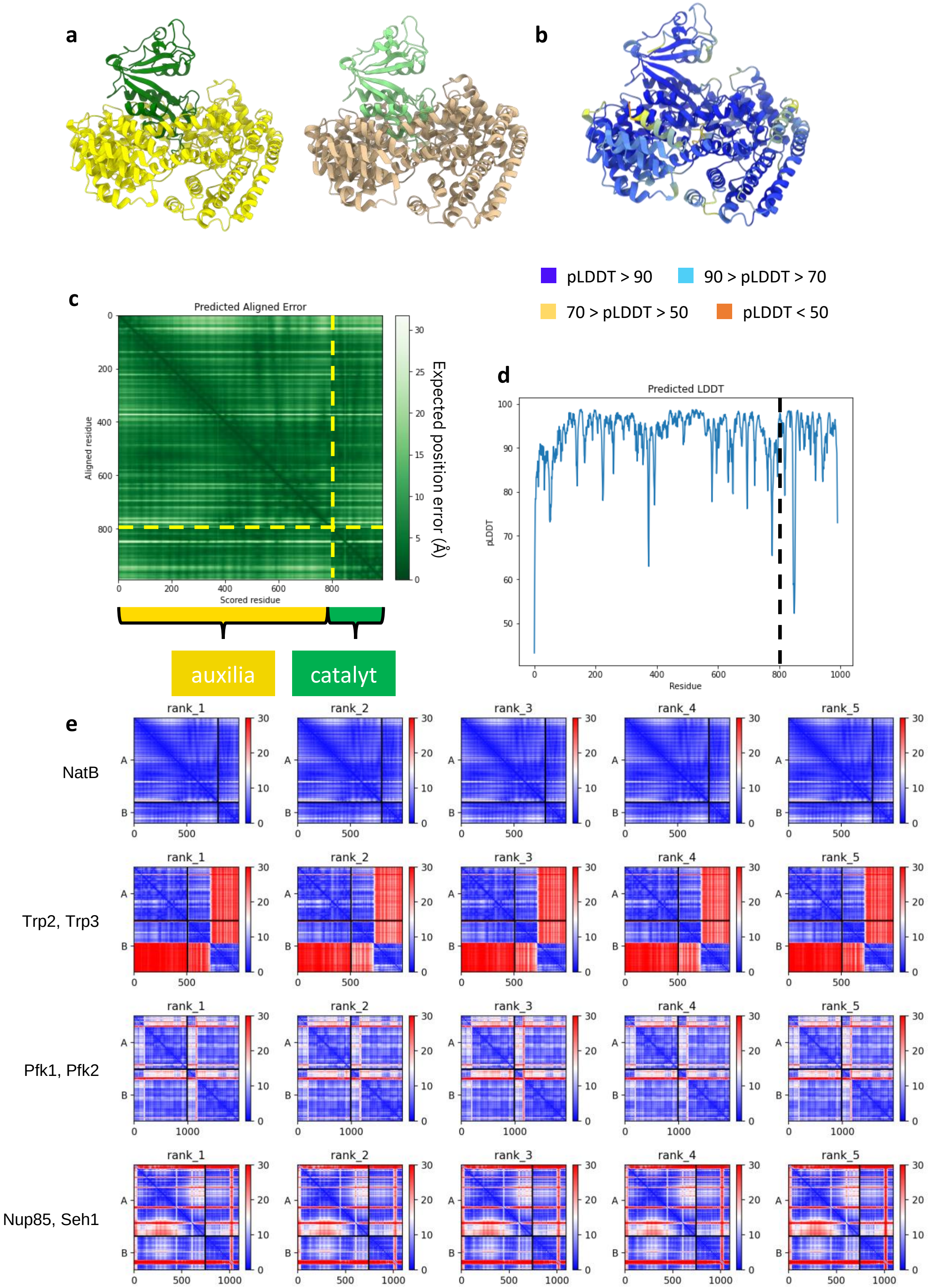

a
b
pLDDT > 90
90 > pLDDT > 70
70 > pLDDT > 50
pLDDT < 50
c
Predicted Aligned Error
Aligned residue
Scored residue
Expected position error (Å)
auxilia
catalyt
d
Predicted LDDT
pLDDT
Residue
e
NatB
Trp2, Trp3
Pfk1, Pfk2
Nup85, Seh1
rank_1
rank_2
rank_3
rank_4
rank_5

f

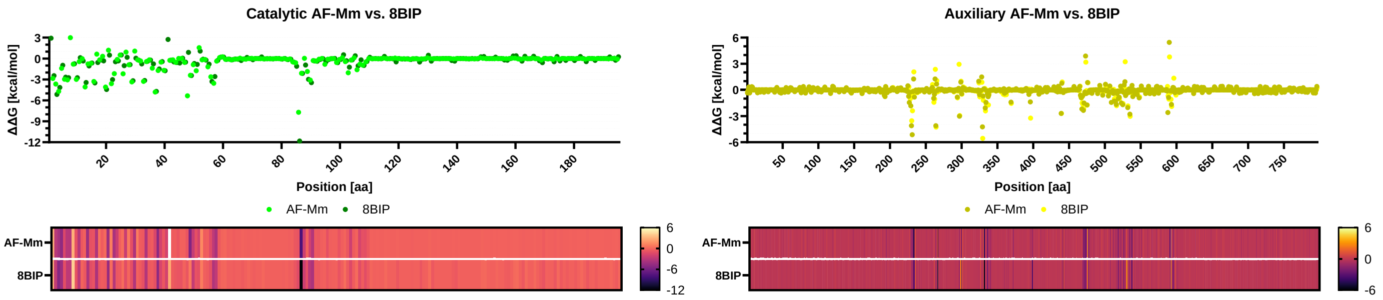

Supplementary Figure 4: NatB structural analysis of AlphaFold-Multimer vs. Cryo-EM

(a) Comparison of a model generated with AlphaFold-Multimer, on the left, and a solved structure of *Saccharomyces cerevisiae* NatB (PDB 8BIP), on the right. Colored in green are the catalytic subunits and the auxiliary subunits are in tan (8BIP) or yellow (AF-Mm model). RMSD between the complexes is less than 1 Å (RMSD between 776 pruned atom pairs is 0.727 Å, or 0.953 Å across all 796 pairs).

(b) pLDDT score per residue overlaid on the AlphaFold Multimer-generated model of *S. cerevisiae* NatB. This model has a very high ipTM (interface predicted TM-score) score of 0.93 with pTM = 0.933, and a pLDDT (per residue confidence score) of each subunit being higher than 92 (model confidence = 0.8 · ipTM + 0.2 · pTM = 0.931).

(c) The predicted aligned error of the entire complex shows a low error for the distance of residues including between the two subunits.

(d) pLDDT score per residue along the protein.

(e) Predicted aligned error of all AlphaFold-Multimer models, ordered by rank for each complex. The color at (x, y) indicates AlphaFold's expected position error at residue x if the predicted and true structures were aligned on residue y, from high error in red (30) to blue (0) for low error. For all models, the error is overall low for each protein's relative alignment with itself. The error is also low at the interface between the proteins. Black lines separate the subunits of the same complex in the same order as indicated on the left. If the PAE is generally low for residue pairs x-y from two different domains, it indicates that AlphaFold predicts well-defined relative positions and orientations for them.

(f) Comparison of the energy profiles of NatB's subunits – AlphaFold-Mm-generated versus a solved structure (PDB: 8BIP). Left: a comparison of the catalytic subunit; Right: a comparison of the auxiliary subunit.

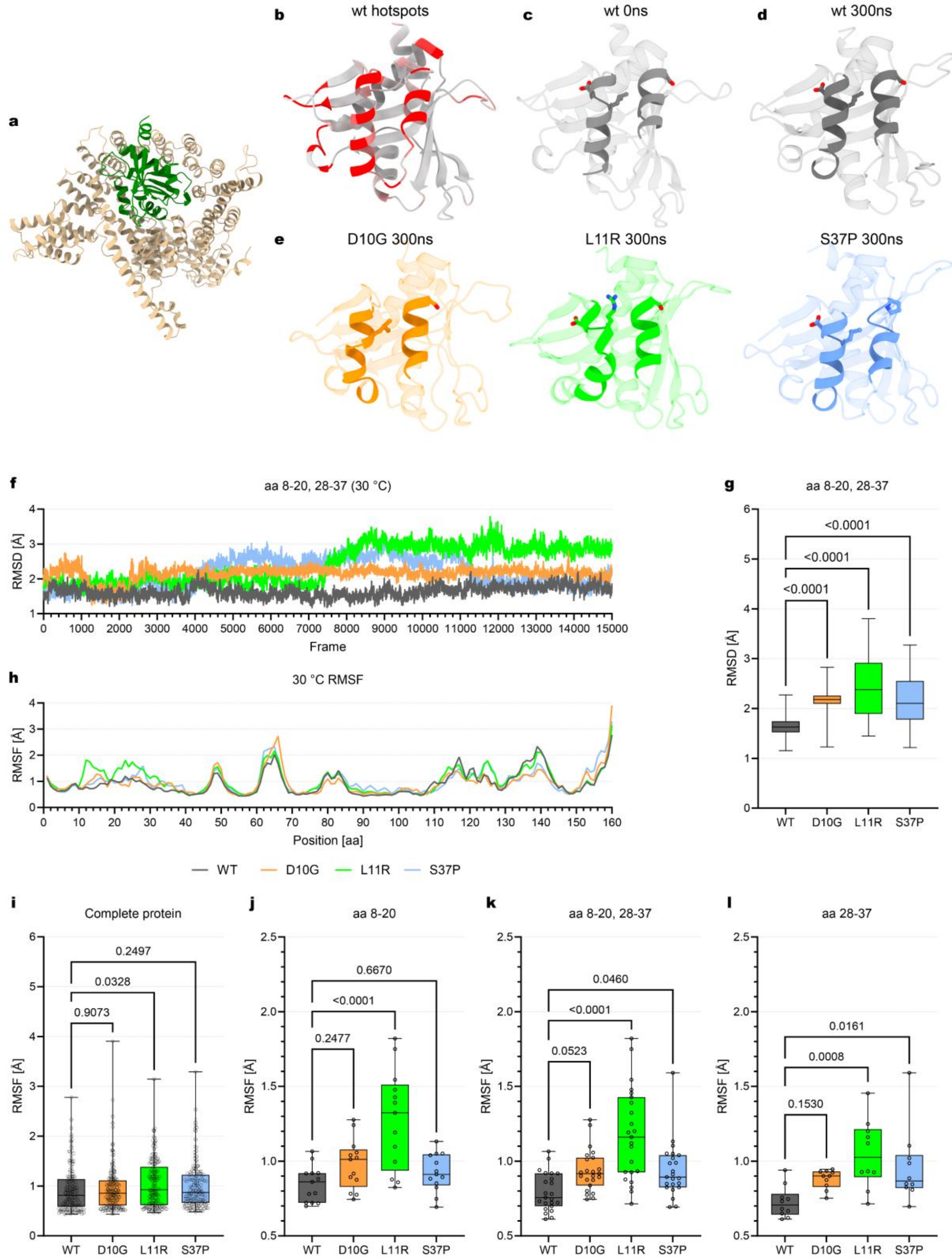

a
b
wt hotspots
c
wt 0ns
d
wt 300ns
e
D10G 300ns
L11R 300ns
S37P 300ns
f
aa 8-20, 28-37 (30 °C)
RMSD [Å]
Frame
g
aa 8-20, 28-37
<0.0001
<0.0001
<0.0001
RMSD [Å]
WT
D10G
L11R
S37P
h
30 °C RMSF
RMSF [Å]
Position [aa]
WT
D10G
L11R
S37P
i
Complete protein
0.2497
0.0328
0.9073
RMSF [Å]
j
aa 8-20
0.6670
<0.0001
0.2477
RMSF [Å]
k
aa 8-20, 28-37
0.0460
<0.0001
0.0523
RMSF [Å]
l
aa 28-37
0.0161
0.0008
0.1530
RMSF [Å]

Supplementary Figure 5: Human Naa10 disease mutants' MD structural analysis reveals a significant impact on alpha helices harboring predicted interface hotspots.

(a) A model of human NatA heterodimer generated by AlphaFold. The catalytic subunit Naa10 is in green, and the auxiliary subunit is in tan.

(b) Human Naa10 from the solved structure PDB: 6C9M[2], highlighting complex interface hotspots in red. NatA complex interface energy contribution per residue in a range of -2 (red) – 0 (grey) ΔΔG [kcal/mol], computed from 300 ns MD simulations, using 1000 frames from the last 20ns, with pyDock bindEy. α-helices at positions: aa 8-20 and aa 28-37, centered, identified as clustering residue hotspots contributing the most energy to the interface.

(c) Wildtype human Naa10 highlighting disease mutants D10G, L11R and S37P (stick representation). All residues are located at the two highly energetic helices.

(d-e) Free human Naa10 subunit thermostability in wildtype **(d)** compared to disease mutants **(e)** D10G, L11R, and S37P. Conformational changes predicted by MD simulations at 30°C, over 300 ns timeframe. Timepoint 0 ns of the simulations as displayed in **(c)**. Only the frame at 300 ns is shown for all. The mutated proteins were obtained by replacing the wildtype residues and equilibration before running production. The MD simulations show the mutants impact the conformation of the two α-helices harboring many hotspots.

(f-g) RMSD for wildtype, D10G, L11R, and S37P computed for the two α-helices. The RMSD boxplots **(g)** indicate that the conformation of the wildtype is maintained during the simulation while the mutants change conformation relative to the starting point (unpaired two-sample t-test).

(h) RMSF of the wildtype and the mutants, per residue, along the ORF.

(i-l) RMSF boxplots of the entire protein or the indicated segments, demonstrating the higher fluctuations of the mutants at the two α-helices (unpaired two-sample t-test).

Supplementary Table 1. TM-Align core regions by residue number along the ORF.

Catalytic subunit	*ScNatA - 6HD5/U*	*ScNatB AF Q06504*	*CaNatB 5k18/B*
TM-Align	1-52,89-102,107-196	1-146,161-173	1-141,153-165
Core w/o IDRs	1-49,89-99,112-175,187-193	1-49,57-67,76-139,165-170	1-48,51-61,72-133,156-161
Minimal region	140	104	102

Auxiliary subunit	*ScNatA 6HD5/t*	*ScNatB AF Q12387*	*CaNatB 5K18/A*
Core/Minimal region	96-350	109-404	76-312

Supplementary Table 2. SSb1/2 Chaperone binding sites of *S. cerevisiae* NatA and NatB subunits (data derived from accession code GSE93830) and TRiC/CCT (from Stein et al. (2019)[1]).

Protein	*Binding Positions (codon/aa)*	*Total*
Naa15	50-59, 260-277, 340-359, 360-377, 471-486, 564-579, 599-605, 623-631, 638-644, 772-780	120
Naa10	151-191	40
Naa25	63-74, 189-206, 270-276, 318-329, 407-416, 729-738	63
Naa20	96-112, 118-125, 127-134, 136-162, 143-154	67
Naa10 TRiC/CCT	103-109	7

Supplementary Table 3. Ribosome binding sites - experimental and predicted binding sites in auxiliary unit. The binding residues of 6HD7 are C_{α}-atoms within 4 Å from any heavy atoms of the ribosome.

PDB	*Site 1 [aa]*	*Site 2 [aa]*	*Site 3 [aa]*	*Site 4 [aa]*
ScNatA (6HD7)	35-49	173-187,212,225	644-656	
CaNatB (5K18)		129-141	579-599,607-643,667-701	299-303,270-271
ScNatB model			489-496,559-567	325,382-389

Supplementary Table 4. pyDock-calculated binding free energies of all complexes, parts, and mutants. pyDock energy in [kcal/mol] by contributions from electrostatic, desolvation, and van der Waals energy, for the *S. cerevisiae* NatA and NatB, and *C. albicans* NatB – as generated by AlphaFold-Multimer and by pyDock followed by long molecular dynamics (1.7 µs). The loops in *Sc*NatA provide more than half of the binding free energy. Both construction methods of the *Sc*NatB model, AlphaFold-Multimer and pyDock followed by MD, provided similar results in terms of the binding free energy.

Structure	*NAT*	*Strain*	*Electrostatics*	*Desolvation*	*Van der Waals*	*Total [kcal/mol]*
6HD5	*Sc*NatA	WT	-131.6	-27.7	-34.0	-211.2
6HD5	*Sc*NatA	R354A, R355A	-137.7	-28.5	-43.3	-203.6
6HD5	*Sc*NatA	R354E, R355E	-131.9	-10.4	-45.2	-178.6
6HD5	*Sc*NatA	WT w/o loops	-37.0	-46.0	-23.1	-106.2
8BIP	*Sc*NatB	WT	-51.8	-39.8	-26.5	-118.2
5K18	*Ca*NatB	WT	-49.5	-4.4	-25.8	-79.7
AlphaFold2 + PyDock	*Sc*NatB	WT	-13.3	-22.6	-33.0	-32.6
AlphaFold2 + PyDock + MD	*Sc*NatB	WT	-54.9	-33.4	-28.8	-117.2
AlphaFold-Multimer	*Sc*NatB	WT	-55.6	-32.8	-31.0	-119.2

Supplementary Table 5. Scores of the highest-ranking model for each complex.

Complex	*pLDDT*	*pTM*	*ipTM*
NatB (Q12387, Q06504)	95.9	0.945	0.949
Trp2, Trp3 (P00899, P00937)	90.7	0.734	0.892
Pfk1, Pfk2 (P16861, P16862)	90	0.916	0.913
Nup85, Seh1 (P46673, P53011)	85.8	0.85	0.887

Supplementary Tables 6-9. Interface energy contribution (ΔΔG [kcal/mol]) per residue in each subunit, as calculated by MM-PBSA along the last 20 ns of the 300 ns simulations. 500 evenly spaced frames were sampled. PDBs with missing atoms and residues were completed as described in the methods. *Sc*NatB was modeled by AlphaFold-Multimer.

Supplementary Table 6. *S. cerevisiae* NatA hotspots contributing <-2 [kcal/mol] to interface formation.

	AUXILIARY				*CATALYTIC*			
	Residue	Position [aa]	AVG	STD	Residue	Position [aa]	AVG	STD
1	ARG	265	-15.38	2.98	ASP	82	-6.22	1.24
2	ARG	354	-7.9	5.64	ARG	7	-4.89	1.36
3	LYS	300	-4.92	2.35	ASP	71	-4.38	3.12
4	ARG	355	-4.68	2.32	ILE	79	-3.86	1.47
5	PHE	453	-4.28	0.79	LEU	52	-3.74	1.2
6	TYR	199	-4.13	1.87	LYS	31	-3.58	2.29
7	PHE	275	-3.64	0.71	ILE	5	-3.56	1.22
8	LYS	295	-3.53	3.27	MET	30	-3.4	0.68
9	PRO	342	-3.33	0.88	ARG	122	-3.38	3.09
10	GLU	419	-3.31	3.48	ILE	10	-3.21	0.68
11	PRO	306	-3.3	0.94	LEU	24	-3.16	0.62
12	ARG	270	-3.13	4	LEU	81	-2.97	0.6
13	LYS	269	-3.02	3.41	MET	127	-2.88	0.72
14	PRO	602	-2.97	0.59	CYS	54	-2.79	2.09
15	ARG	452	-2.94	1.95	ILE	14	-2.76	0.55
16	LYS	258	-2.87	1.88	ASP	12	-2.75	0.34
17	ARG	586	-2.76	0.6	ILE	228	-2.65	0.75
18	ALA	343	-2.5	0.72	MET	34	-2.58	0.69
19	LYS	292	-2.48	1.97	LEU	38	-2.46	0.87
20	ASN	274	-2.42	1.06	ARG	159	-2.39	2.27
21	VAL	341	-2.41	0.75	ILE	3	-2.26	0.65
22	GLU	309	-2.34	2.8	ILE	229	-2.26	1.79
23	LYS	358	-2.34	2.15	THR	205	-2.24	1.14
24	PHE	304	-2.33	1.3	LEU	224	-2.21	1
25	LYS	240	-2.28	0.59	GLU	59	-2.06	1.5
26	LYS	276	-2.25	1.35	ASN	27	-2.02	2.64
27	ILE	262	-2.24	0.77				
28	LYS	356	-2.13	2.8				
29	PHE	575	-2.12	0.61				
30	GLU	502	-2.01	0.78				

Supplementary Table 7. *S. cerevisiae* NatB hotspots contributing <-2 [kcal/mol] to interface formation.

	AUXILIARY				*CATALYTIC*			
	Residue	Position [aa]	AVG	STD	Residue	Position [aa]	AVG	STD
1	TYR	231	-5.14	1.1	ARG	86	-7.71	2.37
2	PRO	329	-4.23	1.11	GLU	48	-5.35	2.94
3	LEU	230	-4.11	0.76	ILE	37	-4.8	0.82
4	TYR	264	-4.1	0.88	ILE	4	-4.71	0.9
5	ASN	297	-3.01	1.98	LEU	20	-4.09	0.79
6	MET	535	-2.78	0.7	PHE	13	-4.07	0.91
7	PHE	439	-2.7	1.02	THR	3	-3.66	1.26
8	ASN	588	-2.59	1.27	ILE	22	-3.6	0.9
9	VAL	470	-2.35	0.7	HIE	57	-3.55	2
10	LYS	468	-2.18	1.39	LEU	29	-3.34	0.57
11	ILE	333	-2.07	0.57	ASN	16	-3.29	1.77
12	MET	532	-2.03	0.68	PHE	33	-3.27	0.97
13					ILE	90	-3.09	0.38
14					PHE	7	-3.05	0.74
15					VAL	10	-2.99	0.66
16					PRO	6	-2.97	0.61
17					THR	2	-2.86	1.92
18					LYS	56	-2.62	1.67
19					MET	49	-2.41	0.76
20					ASN	26	-2.19	1.57
21					LEU	23	-2.17	0.95
22					PHE	87	-2.12	0.66
23					MET	103	-2.04	0.9

Supplementary Table 8. *C. albicans* NatB hotspots contributing <-2 [kcal/mol] to interface formation.

	AUXILIARY				*CATALYTIC*			
	Residue	Position [aa]	AVG	STD	Residue	Position [aa]	AVG	STD
1	ARG	272	-4.7	2.57	SER	3	-6.33	0.87
2	ARG	496	-4.04	2.49	LEU	20	-4.5	0.9
3	ARG	433	-3.28	2.53	VAL	29	-4.48	0.95
4	TYR	404	-3.11	1.64	ASN	16	-4.43	1.5
5	PHE	490	-2.94	0.54	ILE	37	-4.11	0.55
6	LEU	208	-2.89	0.51	PHE	13	-4.05	0.75
7	PHE	493	-2.76	0.65	SER	30	-3.48	2.07
8	LEU	304	-2.41	0.57	PHE	7	-3.46	1.01
9	PHE	240	-2.32	0.7	ILE	87	-3.22	0.4
10	MET	434	-2.04	0.62	PRO	22	-2.75	0.76
11	PHE	243	-2.01	0.63	ARG	86	-2.36	3.21
12	VAL	483	-2	0.33	GLU	48	-2.3	2.91
13					ASN	28	-2.08	2.14
14					GLN	41	-2.08	1.29

Supplementary Table 9. *H. sapiens* Naa10 hotspots contributing <-2 [kcal/mol] to interface formation.

	CATALYTIC			
	Residue	Position [aa]	AVG	STD
1	PHE	32	-9.56	2.74
2	ARG	79	-8.92	3.26
3	GLU	48	-7.27	1.83
4	LYS	29	-7.1	2.65
5	HID	16	-6.8	1.22
6	LEU	36	-6.26	1.58
7	PHE	102	-5.92	1.63
8	TYR	33	-4.89	2.55
9	LEU	20	-4.6	2.54
10	MET	12	-3.36	1.51
11	ASP	47	-3.34	1.56
12	PRO	23	-3.27	0.95
13	LEU	19	-3.08	0.93
14	GLU	9	-2.49	1.15
15	PRO	8	-2.41	1
16	MET	28	-2.25	0.55
17	GLU	46	-2.23	0.56
18	ARG	82	-2.16	1.56

Supplementary Table 10.
pyDock bindEy energy contributions [kcal/mol] from each component for 6HD5 at four time points at 25 °C. 300 ns long simulations

Complex	*Time*	*Electrostatic*	*Desolvation*	*Van der Waals*	*Total [kcal/mol]*
WT	0.0	-123.5	-38.9	-36.2	-198.6
WT	100.0	-164.2	-0.6	-48.6	-213.4
WT	200.0	-176.1	-5.5	-48.8	-230.4
WT	300.0	-176.2	22.0	-48.3	-202.5
WT	**Average**	**-160.0**	**-5.7**	**-45.4**	**-211.2**
AA	0.0	-98.6	-44.8	-37.0	-180.5
AA	100.0	-151.4	-18.4	-45.7	-215.6
AA	200.0	-141.7	-20.6	-44.0	-206.4
AA	300.0	-147.9	-14.5	-49.4	-212.0
AA	**Average**	**-134.9**	**-24.5**	**-44.0**	**-203.6**
EE	0.0	-87.2	-40.6	-38.3	-166.2
EE	100.0	-134.3	-5.4	-42.5	-182.3
EE	200.0	-125.3	-8.4	-43.8	-177.6
EE	300.0	-126.7	-20.0	-41.6	-188.4
EE	**Average**	**-118.3**	**-18.6**	**-41.5**	**-178.6**

Supplementary Table 11. pyDock bindEy energy contributions in [kcal/mol] from each mutated residue of 6HD5 at four time points at 25 °C along 300 ns simulation.

*Sc*Naa10 WT

Time [ns]	0	100	200	300	Average
R354 [kcal/mol]	-6.7	-6.7	-3.1	-5.9	-5.6
R355 [kcal/mol]	-14.1	-7.2	-7.9	-11.1	-10.1
Total [kcal/mol]	-198	-213	-230	-202	-211

*Sc*Naa10 mutant R354E, R355E

Time [ns]	0	100	200	300	Average
R354 [kcal/mol]	4.2	3.4	5.2	1.1	3.5
R355 [kcal/mol]	8.4	2.5	8.5	4.4	5.9
Total [kcal/mol]	-166	-182	-177	-188	-178

*Sc*Naa10 mutant R354A, R355A

Time [ns]	0	100	200	300	Average
R354 [kcal/mol]	-0.7	-1.1	-1.3	-0.4	-0.9
R355 [kcal/mol]	-0.8	-1.6	-0.2	-0.2	-0.7
Total [kcal/mol]	-180	-215	-206	-212	-203

Supplementary Table 12. List of strains used.

Strain	*Genotype*	*Source*
BY4741	MATa, his3Δ1, leu2Δ0, met15Δ0, ura3Δ0	Euroscarf
yAS6	BY4741, NAA25-GFP:: HisMX	This study
yAS7	BY4741, NAA15-GFP:: HisMX	This study
yAS9	BY4741, NAA20-GFP:: kanMX4	This study
yAS12	BY4741, NAA10-GFP:: kanMX4	This study
yAS17	BY4741, NAA10-GFP::kanMX4, naa15Δ::hphNT1	This study
yAS20	BY4741, NAA15-GFP:: HisMX, naa10Δ::hphNT1	This study
yAS21	BY4741, NAA20-GFP::kanMX4, naa25Δ::hphNT1	This study
yAS26	BY4741, NAA25-GFP:: HisMX, naa20Δ::hphNT1	This study
yAS49	mCherry-NAA15	This study
yAS50	mCherry-NAA15; *naa10*Δ	This study
yAS66	mCherry-NAA15,R354A R355A NAA15	This study
yAS69	mCherry-NAA15,R354E R355E NAA15	This study
yAS74	2PA, 5IA, 7RA NAA10	This study

Supplementary Table 13. List of primers used for qPCR.

Gene	*Sequence*	*Strand*
NAA15	CGGCAGCTTTGAACAATGGG	Forward
NAA15	GATGTCCAGTTGGCACGGTA	Reverse
ACT1	CTGCCGGTATTGACCAAACT	Forward
ACT1	CGGTGATTTCCTTTTGCATT	Reverse

Supplementary Table 14. List of gRNA (guide RNA) primers for CRISPR\Cas9.

Gene	*Sequence*	*gRNA primers*
NAA15	ACCCTAACGAAGTATGTCTGTTTT	Forward
NAA15	AGACATACTTCGTTAGGGTGATCA	Reverse
NAA15	TTACCAAAGAAGGAAGTCCAGTTTT	Forward
NAA15	TGGACTTCCTTCTTTGGTAAGATCA	Reverse
NAA15	AGAAAATGACCAGTTCCTCGGTTTT	Forward
NAA15	CGAGGAACTGGTCATTTTCTGATCA	Reverse
NAA15	AGAAAATGACCAGTTCCTCGGTTTT	Forward
NAA15	CGAGGAACTGGTCATTTTCTGATCA	Reverse
NAA10	TCATATTCTCTCGTGGCCAGGTTTT	Forward
NAA10	CTGGCCACGAGAGAATATGAGATCA	Reverse

Supplementary Table 15. List of primers for dDNA (donor DNA) for CRISPR\Cas9.

Gene	*Sequence*	*gRNA primers*
NAA15	TTAAGACAAATACCATTGAGGAAGGCGATTGACCCTAACGAAGTATG GTGAGCAAGGGCGAGGA	Forward
NAA15	CTATTTTAGCTGCTGGCTTGGGCTTAGTACTTCTTTTCCTAGATCCTGC TCCTGCTCCTGCCTTGTACAGCTCGTCCATG	Reverse
NAA15	TCCAGCAACTTTTTCCAACGTGAAACCCCTTTACCAAGCAGCTAAGTC CAAGGTTTCACCACTATTGGAGAAAATTGTCC	Forward
NAA15	GGACAATTTTCTCCAATAGTGGTGAAACCTTGGACTTAGCTGCTTGGT AAAGGGGTTTCACGTTGGAAAAAGTTGCTGGA	Reverse
NAA15	TCCAGCAACTTTTTCCAACGTGAAACCCCTTTACCAAGAAGAGAAGT CCAAGGTTTCACCACTATTGGAGAAAATTGTCC	Forward
NAA15	GGACAATTTTCTCCAATAGTGGTGAAACCTTGGACTTCTCTTCTTGGT AAAGGGGTTTCACGTTGGAAAAAGTTGCTGGA	Reverse
NAA10	ACATACGATCAAGCTCCAAAATAAAACTTCGTCAACCATGGCTATTA ATGCTCGCGCAGCGACAATCAATGACATTATAT	Forward
NAA10	CTTCACAGTCCAAAGTGGTGGTAGTAGCAACAAACGAAGCTTCAGGC CATGACAGAATATGATACATATAATATTTCATC	Reverse

Supplementary Table 16. Molecular dynamics checklist.

**Reliability and reproducibility checklist for molecular dynamics simulations**<br>***All boxes must be marked YES by acceptance unless an N/A option is available**	**Yes**	**N/A**	**Response**<br>**(Please state where this information can be found in the text)**
**1. Convergence of simulations and analysis**			
1a. Is an evaluation presented in the text to show that the property being measured has equilibrated in the simulations (*e.g.* time-course analysis)?	☒		All RMSF calculations were done at thermodynamic equilibrium, as validated by the root mean square deviation (RMSD), as shown in supplementary fig. 1
1b. Then, is it described in the text how simulations are split into equilibration and production runs and how much data were analyzed from production runs?	☒		Equilibration consisted of a total of 8 ns of MD.<br>We computed MMPBSA interaction energy values per residue for 500 conformations from the last 20 ns of the 300 ns long production simulations. As described in the methods section.
1c. Are there at least 3 simulations per simulation condition with statistical analysis?	☒		Yes.
1d. Is evidence provided in the text that the simulation results presented are independent of initial configuration?	☒		The 3 repeats were used to show independence from initial conditions.
**2. Connection to experiments**			
2a. Are calculations provided that can connect to experiments (*e.g.* loss or gain in function from mutagenesis, binding assays, NMR chemical shifts, J-couplings, SAXS curves, interaction distances or FRET distances, structure factors, diffusion coefficients, bulk modulus and other mechanical properties, *etc*.)?	☒		Fig. 3d,f,g,h
**3. Method choice**			
3a. Is it described in the text what force field and water model are used and why?	☒		ff19SBonlysc forcefield for proteins and OPC3BOX forcefield was used to soak the complex. We used this force field combination since:<br><br>From: Amber 2021<br>DA Case, HM Aktulga, K Belfon, I Ben-Shalom… - 2021 - “Our results [19] showed that ff19SB pairs best with the more

			accurate water model OPC [20], and that the older TIP3P model”
3b. Do simulations contain membranes, membrane proteins, intrinsically disordered proteins, glycans, nucleic acids, polymers, or cryptic ligand binding?	☐	☒	Response not needed if **N/A**
If 3b is **YES**, are enhanced sampling methods used?	☐	☒	Response not needed if **N/A**
If enhanced sampling methods are used, are the convergence criteria clearly stated?	☐		
If 3b is **YES**, is it explained in the text why or why not enhanced sampling methods are used?	☐		
**4. Code and reproducibility**			
4a. Is a table provided describing the system setup, such as simulation box dimensions, total number of atoms, total number of water molecules, salt concentration, lipid composition (number of molecules and type)?	☒		Salt concentration [NaCl]=0.15M.<br>We used truncated octahedron box, with a minimum distance of 12 Angstroms between any protein atom and the closest edge of the box (this produces boxes with different sizes according to the system, which include between 10000 and 45000 water molecules).
4b. Is it described in the text what simulation and analysis software and which versions are used?	☒		Yes.<br>AmberTools19, cpptraj, Amber18 with pmemd.CUDA version.
4c. Are initial coordinate and simulation input files and a coordinate file of the final output provided as supplementary files or in a public repository?	☒		Initial coordinate and simulation input files are now provided. No final output coordinate file is provided since no analysis or calculations were done on such files.
4d. Is there custom code or custom force field parameters?	☐	☒	Response not needed if **N/A**
If **YES**, are they provided as supplementary profiles or in a public repository?	☐		

Supplementary References

1. Stein, K. C., Kriel, A. & Frydman, J. Nascent Polypeptide Domain Topology and Elongation Rate Direct the Cotranslational Hierarchy of Hsp70 and TRiC/CCT. *Mol Cell* **75**, 1117-1130.e5 (2019).
2. Gottlieb, L. & Marmorstein, R. Structure of Human NatA and Its Regulation by the Huntingtin Interacting Protein HYPK. *Structure* **26**, 925-935.e8 (2018).
