# Supplementary material for: Diverging co-translational protein complex assembly pathways are governed by interface energy distribution
Source: Nat Commun. 2024 Mar 25;15:2638. doi: 10.1038/s41467-024-46881-w (PMC10963368; doi:10.1038/s41467-024-46881-w)
Supplement: Supplementary file 3 — Description of Additional Supplementary Files [file 41467_2024_46881_MOESM3_ESM.pdf]

Description of Additional Supplementary Files

File name: Supplementary Movie 1
Description: related to Figure 1 - A video showing 300 ns long MD simulation of *S. cerevisiae* Naa10, *S. cerevisiae* Naa20, and *C. albicans* Naa20 (left to right) at 100 C, in complex (top) and free (bottom).

File name: Supplementary Movie 2
Description: related to Figure 1 - A video showing 300 ns long MD simulation of *S. cerevisiae* Naa15, *S. cerevisiae* Naa25, and *C. albicans* Naa25 (left to right) at 100 C, in complex (top) and free (bottom).

File name: Supplementary Movie 3
Description: related to Figure 3 - A simulation of the system time evolution of the wildtype and the two mutants (R354A, R355A, and R354E, R355E). The simulation is of the entire complex but shows a zoomed in window of the mutation and its interacting partner's residues. While the mutation to alanine behaves like the wildtype, a strong repulsion can be seen in the mutation to glutamates, impairing interface formation.
