# Supplementary material for: Diverging co-translational protein complex assembly pathways are governed by interface energy distribution
Source: Nat Commun. 2024 Mar 25;15:2638. doi: 10.1038/s41467-024-46881-w (PMC10963368; doi:10.1038/s41467-024-46881-w)
Supplement: Supplementary file 7 — Reporting Summary [file 41467_2024_46881_MOESM7_ESM.pdf]

nature portfolio

Corresponding author(s): Ayala Shiber

Last updated by author(s): Feb 20, 2024

# Reporting Summary

Nature Portfolio wishes to improve the reproducibility of the work that we publish. This form provides structure for consistency and transparency in reporting. For further information on Nature Portfolio policies, see our Editorial Policies and the Editorial Policy Checklist.

## Statistics

For all statistical analyses, confirm that the following items are present in the figure legend, table legend, main text, or Methods section.

| n/a | Confirmed | |
|---|---|---|
| ☐ | ☒ | The exact sample size (*n*) for each experimental group/condition, given as a discrete number and unit of measurement |
| ☐ | ☒ | A statement on whether measurements were taken from distinct samples or whether the same sample was measured repeatedly |
| ☐ | ☒ | The statistical test(s) used AND whether they are one- or two-sided<br>*Only common tests should be described solely by name; describe more complex techniques in the Methods section.* |
| ☒ | ☐ | A description of all covariates tested |
| ☒ | ☐ | A description of any assumptions or corrections, such as tests of normality and adjustment for multiple comparisons |
| ☐ | ☒ | A full description of the statistical parameters including central tendency (e.g. means) or other basic estimates (e.g. regression coefficient) AND variation (e.g. standard deviation) or associated estimates of uncertainty (e.g. confidence intervals) |
| ☐ | ☒ | For null hypothesis testing, the test statistic (e.g. *F*, *t*, *r*) with confidence intervals, effect sizes, degrees of freedom and *P* value noted<br>*Give P values as exact values whenever suitable.* |
| ☒ | ☐ | For Bayesian analysis, information on the choice of priors and Markov chain Monte Carlo settings |
| ☒ | ☐ | For hierarchical and complex designs, identification of the appropriate level for tests and full reporting of outcomes |
| ☐ | ☒ | Estimates of effect sizes (e.g. Cohen's *d*, Pearson's *r*), indicating how they were calculated |

*Our web collection on statistics for biologists contains articles on many of the points above.*

## Software and code

Policy information about availability of computer code

Data collection

Imaging was done using Nikon NIS-Elements 5.21.03 (Build 1489)

Data analysis

Andor Technology Limited – Imaris v9.3.1, https://imaris.oxinst.com/
cutadapt v1.8.3, https://pypi.org/project/cutadapt/
Bowtie2 v2.2.5.0 Langmead and Salzberg, 2012 http://bowtie-bio.sourceforge.net/bowtie2/index.shtµl
Tophat2 v2.0.13 Kim et al., 2013 http://ccb.jhu.edu/software/tophat/downloads/
Python v2.7 and v3.4 Python Software Foundation, https://www.python.org/downloads/
ImageJ software, v1.50i, https://imagej.nih.gov/ij/
TM-Align v, https://zhanggroup.org/TM-align/
Visual Molecular Dynamics v1.9, https://www.ks.uiuc.edu/Research/vmd/
UCSF ChimeraX v1.6, https://www.cgl.ucsf.edu/chimerax/
AmberTools19 with ff19SBonlysc, https://ambermd.org/AmberTools.php
CPPTRAJ, https://amberhub.chpc.utah.edu/cpptraj/
pyDock, https://life.bsc.es/pid/pydock/
OPRA, https://life.bsc.es/pid/opra
GraphPad Prism v10.0.0, https://www.graphpad.com
Trans Proteomic Pipeline v6.1 http://www.tppms.org/
Comet 2023.01 rev. 2 https://comet-ms.sourceforge.net/
PeptideProphet https://peptideprophet.sourceforge.net/

For manuscripts utilizing custom algorithms or software that are central to the research but not yet described in published literature, software must be made available to editors and reviewers. We strongly encourage code deposition in a community repository (e.g. GitHub). See the Nature Portfolio guidelines for submitting code & software for further information.

## Data

Policy information about availability of data

All manuscripts must include a data availability statement. This statement should provide the following information, where applicable:

- Accession codes, unique identifiers, or web links for publicly available datasets
- A description of any restrictions on data availability
- For clinical datasets or third party data, please ensure that the statement adheres to our policy

The translatomics data generated in this study have been deposited in the Sequence Read Archive repository under accession code: PRJNA1030163 [http://www.ncbi.nlm.nih.gov/bioproject/1030163]. Figure 1 also relies on raw data derived from the Gene Expression Omnibus repository: GSE116570. Figure 2 also relies on raw data derived from GSE93830. Figure 4 also relies on raw data derived from GSE116570, PRJEB46361, and PRJEB50305. The MS data generated in this study have been deposited in the ProteomeXchange Consortium with identifier PXD048082 [http://proteomecentral.proteomexchange.org/cgi/GetDataset?ID=PXD048082].
PDB: 6HD5, 6HD7, 5K18, and 8BIP.
All other data are available from the corresponding authors upon reasonable request.

## Research involving human participants, their data, or biological material

Policy information about studies with human participants or human data. See also policy information about sex, gender (identity/presentation), and sexual orientation and race, ethnicity and racism.

| | |
|---|---|
| Reporting on sex and gender | N/A |
| Reporting on race, ethnicity, or other socially relevant groupings | N/A |
| Population characteristics | N/A |
| Recruitment | N/A |
| Ethics oversight | N/A |

Note that full information on the approval of the study protocol must also be provided in the manuscript.

# Field-specific reporting

Please select the one below that is the best fit for your research. If you are not sure, read the appropriate sections before making your selection.

☒ Life sciences ☐ Behavioural & social sciences ☐ Ecological, evolutionary & environmental sciences

For a reference copy of the document with all sections, see nature.com/documents/nr-reporting-summary-flat.pdf

# Life sciences study design

All studies must disclose on these points even when the disclosure is negative.

| | |
|---|---|
| Sample size | For aggregation assay by microscopy, sample size was determined by statistical power of the test, given expected differences, and we opted for higher statistical power.<br>Based on the assumption of two normally distributed groups, and assuming a difference of at least 20%, according to t-test for independent groups and a significance of at least 0.05 and power of 0.95, at least 88 samples are required for each group. Therefore, n=150 for microscopy experiments.<br>For SeRP, RIP-qPCR etc. sample size was not determined as parallel sampling of at least ~5.6X10^9 cells (according to O.D measurements at 600nm) were done, as previously published for these experimental approaches in Shiber et al. (2018), Seidel et al (2022). |
| Data exclusions | No data were excluded from the analyses. Minimal detection thresholds were used for including each gene in the analysis (A threshold of 64 total counts per gene was chosen as a point where the inter-replicate variation approached its infinite-counts asymptote and counting statistics contributed little. As in: Ingolia, N.T. et al., Science. 10; 324(5924)(2009)). |
| Replication | We used a minimal of 2 independent biological replicates per experiment. For each specific experiment the number of replicates is indicated in the text or figure legend. The replicates were highly reproducible as indicated by the shaded area between SeRP replicates, indicating the degree of experimental variation. Similarly, imaging stability assays and qPCR results all showed high reproducibility. |
| Randomization | No arbitrary experimental group allocation took place in this study, hence randomization procedures do not apply. |
| Blinding | No arbitrary experimental group allocation took place in this study, hence blinding procedures do not apply. |

# Reporting for specific materials, systems and methods

We require information from authors about some types of materials, experimental systems and methods used in many studies. Here, indicate whether each material, system or method listed is relevant to your study. If you are not sure if a list item applies to your research, read the appropriate section before selecting a response.

## Materials & experimental systems

| n/a | Involved in the study |
|---|---|
| ☐ | ☒ Antibodies |
| ☒ | ☐ Eukaryotic cell lines |
| ☒ | ☐ Palaeontology and archaeology |
| ☒ | ☐ Animals and other organisms |
| ☒ | ☐ Clinical data |
| ☒ | ☐ Dual use research of concern |
| ☒ | ☐ Plants |

## Methods

| n/a | Involved in the study |
|---|---|
| ☒ | ☐ ChIP-seq |
| ☒ | ☐ Flow cytometry |
| ☒ | ☐ MRI-based neuroimaging |

## Antibodies

| | |
|---|---|
| Antibodies used | Lama monoclonal anti-GFP (Döring K, Cell, Jul 13;170(2):298-311.e20. (2017)).<br>Anti-HA [12CA5] recombinant mouse monoclonal antibody (Field, J. Molecular and Cellular Biology, 8:5, 2159-2165 (1988)).<br>Polyclonal antibody HA.11 was raised against the twelve amino acid sequence CYPYDVPDYASL (Bosshart, H. J Cell Biol , 126 (5): 1157–1172 (1994)). |
| Validation | All antibodies were successfully validated as mentioned in their respective papers, see above.<br>Additionally, incubating the antibodies with mock pull downs did not generate detectable chemiluminescent signal, or ribosome-nascent-chain enrichment foot-print signal (data shown in Supplementary Information). |

## Plants

| | |
|---|---|
| Seed stocks | N/A |
| Novel plant genotypes | N/A |
| Authentication | N/A |
